# Do highly ornamented and less parasitized males have high quality sperm? – an experimental test for parasite-induced reproductive trade-offs in European minnow (*Phoxinus phoxinus*)

**DOI:** 10.1002/ece3.1267

**Published:** 2014-10-21

**Authors:** Jukka Kekäläinen, Juhani Pirhonen, Jouni Taskinen

**Affiliations:** 1 Department of Biology, University of Eastern FinlandP.O. Box 111, Joensuu, FI-80101, Finland; 2 Centre for Evolutionary Biology, School of Animal Biology, University of Western AustraliaCrawley, WA, Australia; 3 Department of Biological and Environmental Sciences, University of JyväskyläJyväskylä, Finland

**Keywords:** Fertility, parasite, reproduction, secondary sexual ornamentation, sperm, trade-off

## Abstract

Parasites take their resources from hosts and thus directly reduce available resources for hosts’ own body functions, such as growth and reproduction. Furthermore, parasite infections cause significant indirect costs to their hosts in terms of increased investments on immune defense. In this study, we investigated the impact of parasite infection on the sperm quality and expression of secondary sexual ornamentation (saturation of the red abdominal colouration and number of breeding tubercles) in the Eurasian minnow (*Phoxinus phoxinus*). We exposed minnows to a high and low dose of common nonspecific fish ectoparasite, the glochidia larvae of duck mussel (*Anodonta anatina*) and tested whether parasite infection leads to trade-off in sperm quality and/or ornamental expression. We found that glochidia infection reduces the curvature of the sperm swimming trajectory, number of breeding tubercles, and possibly male competitive ability, but does not affect expression of male color ornamentation. Furthermore, glochidia infection was found to reduce sperm motility, but only when all the noninfected individuals were excluded from the model. Supporting one of the predictions by phenotype-linked fertility hypothesis both in high-infection and low-infection group male breeding colouration was positively associated with sperm quality. Our results suggest that although glochidia infection may have negative impact on male reproductive success, parasite-induced costs may not create strong trade-off between breeding colouration and sperm quality or that such trade-off become detectable only in resource-limited conditions.

## Introduction

According to life-history theory, resources allocated to one trait inevitably reduces amount of available resources to the other(s) (Fabian and Flatt 2012). One of the most important mediators of life-history trade-offs in nature are parasites, which exploit the nutritional resources of their hosts and thus reduce available resources for host growth and reproduction. Parasites also exert strong selection pressures on hosts’ immune defense (Grenfell and Dobson 1995; Sorci et al. 1997; Bonneaud et al. 2003), as both the maintenance of immune system and its activation are energetically expensive to the host (Kraaijeveld and Godfray 1997; Lochmiller and Deerenberg 2000; McNamara et al. 2013). Thus, both parasite resource exploitation (direct costs) and elevated immune-related (indirect) costs are expected to be strongly traded-off against host's other costly life-history traits such as reproduction. Such trade-offs have been demonstrated in number of taxa (Sheldon and Verhulst 1996), including insects (Polak and Markow 1995; but see Drayton et al. 2013), other invertebrates (Lawniczak et al. 2007), bivalves (Taskinen and Saarinen 1999), fish (Kolluru et al. 2009) and birds (Råberg et al. 2000; Bonneaud et al. 2003). In addition, numerous sexual selection studies have shown that resources allocated to immunity and parasite resistance reduce investments in secondary sexual ornamentation, so that only males in good condition may be able to invest resources on both (Zuk and Stoehr 2002; Kerr et al. 2010).

Although these premating trade-offs are relatively well known, much less attention has been paid to similar trade-offs later, after the copulation or mating (Simmons and Roberts 2005; Kerr et al. 2010; Radhakrishnan and Fedorka 2012). In particular, the effect of parasites on sperm quality has been severely neglected (but see Liljedal et al. 1999). This is probably largely dependent on the fact that sperm production has traditionally believed to be cheap. This view has now been challenged and in contrast to original assumption several studies have demonstrated that sperm production costs can in fact be considerable high (Van Voorhies 1992; Olsson et al. 1997; Paukku and Kotiaho 2005; Losdat et al. 2011; Dowling and Simmons 2012; Simmons 2012). This raises the possibility that parasite-induced direct (host resource exploitation) or indirect (immunity) costs may constrain production of high quality ejaculates (Liljedal et al. 1999). Furthermore, immune system treat spermatozoa as nonself (“foreign cells”) and thus spermatogenic cells must be protected via immunosuppression (Meinhard and Hedger 2011). As immune system upregulation during pathogen infections may weaken this protection, reduction in sperm quality may also be a consequence of male autoimmune reactions against own sperm cells.

Life-history trade-offs are expected to occur also between different reproductive traits (e.g., Roff 2002; Mautz et al. 2013). For example, sperm competition theory predicts that males face a trade-off between secondary sexual ornamentation and sperm quality (Parker and Pizzari 2010; Simmons et al. 2011; Parker et al. 2013). On the other hand, phenotype-linked fertility hypothesis (PLFH) predicts positive association between these traits (Sheldon 1994). Earlier studies testing on the PLFH have reported contradictory results: Some studies have found support for the hypothesis (Kortet et al. 2004a; Locatello et al. 2006; Rogers et al. 2008; Janhunen et al. 2009), whereas others have found either no associations between secondary sexual traits and sperm quality or negative correlations (Liljedal et al. 1999; Skinner and Watt 2007; Liljedal et al. 2008; Pitcher et al. 2009; Rowe et al. 2010; Klaus et al. 2011). In their recent meta-analysis, Mautz et al. (2013) found that secondary sexual trait expression was only weakly (positively) associated with sperm viability, but not with other sperm traits (sperm number, swimming velocity, and size), although also these three associations tended to be positive.

Positive associations between ornamental expression and ejaculate quality can theoretically be expected if both secondary sexual traits and ejaculate traits are condition-dependent (Mautz et al. 2013). Carotenoid sexual ornaments have also been hypothesized to signal male's ability to fight against free radical-induced sperm damage as animals use carotenoids and other antioxidants to inactivate free radicals (Blount et al. 2001). Given that sperm is highly susceptible to attack of free radicals (that can severely impair fertility), males with elaborate carotenoid-based ornamentation may have better ability to protect their sperm from oxidative stress, leading to positive association between ornamental expression and sperm quality (Blount et al. 2001; Helfenstein et al. 2010).

In Eurasian minnows (*Phoxinus phoxinus* L.), males develop bright red, carotenoid-based abdominal breeding colouration and keratin-based breeding tubercles during the spawning season. Male breeding colouration has earlier been shown to be negatively associated with parasite (*Philometra ovata* and *Neoechinorhynchus rutili*) abundance (Kekäläinen et al. 2011). Furthermore, male colouration or breeding tubercles have also been demonstrated to predict male dominance status (Jacob et al. 2009; Kekäläinen et al. 2010; see also Kortet et al. 2004b), genetic heterozygosity (Müller and Ward 1995), and swimming performance (Lai et al. 2013). However, it is not known whether parasite infections have impact on male sperm quality or whether male breeding ornamentation could reveal information about male fertilization ability (phenotype-linked fertility hypothesis).

In this study, we investigated these questions by experimentally infecting minnows with high and low dose of duck mussel (*Anodonta anatina* L.) glochidia larvae and by studying the associations between parasite abundance, sperm quality, and male ornamentation. Glochidia of *A. anatina* and other unionid mussels are common generalist parasites of freshwater fish, including *P. phoxinus* (Dartnall and Walkey 1979; Anders and Wiese 1993; Jensen et al. 2001). After being released from females, glochidia larvae attach mainly to the fins and gills of the host fish, where they will be encapsulated by the host epithelial cells (Rogers-Lowery and Dimock 2006). After 2–8 weeks, parasitizing period glochidia metamorphose to young mussels and leave the host fish (Anders and Wiese 1993). However, eventual metamorphosis success is highly dependent on individual and species-specific differences in host immunity (e.g., Douda et al. 2012). Even in suitable host species, most of the initially attached glochidia are often sloughed off by the host immune system before the transformation is complete.

Besides triggering various immune responses in the hosts (Dodd et al. 2005; Rogers-Lowery and Dimock 2006), glochidia infections also reduce host growth, cause respiratory stress, increase host vulnerability to secondary infections by fungi and bacteria (Meyers et al. 1980), and in extreme cases also cause host death (Howerth and Keller 2006). Together these observations suggest that the costs of glochidial infection may be high and thus potentially able to act as an important mediator of life-history trade-offs in their host species. It should also be pointed out that *A. anatina* glochidia release takes place in spring (Taskinen et al. 1997), just before *P. phoxinus* breeding period. Thus, in natural populations where *A. anatina* and *P. phoxinus* co-occur, the infection and developmental period of glochidia in fish coincides with spawning of *P. phoxinus*.

The primary aim of the study was to test the following questions: (1) Is male sperm quality negatively related to parasite numbers (parasite resistance-fertility trade-off)?; (2) Is male secondary sexual ornamentation negatively related to parasite numbers (parasite resistance-ornamentation trade-off)?; and (3) Does secondary sexual ornamentation reveal potential information about male fertility (phenotype-linked fertility hypothesis)?

## Materials and Methods

### Study species and experimental infections

Sexually mature minnows were collected by dip nets from Uuronpuro brook in Eastern Finland (62° 51′ N, 29° 59′ E) on 22 May 2011, at water temperature of 9.0°C. *A. anatina* does not occur in this brook. Accordingly, none of the fish had earlier glochidium infections. Fish were transported to the laboratory of the Konnevesi Research Station (University of Jyväskylä, Finland) where minnows were randomly divided and housed in six 15 L plastic containers (n = ca. 15 males and 10 female/container) with continuous 8.5°C water flow from the nearby Lake Konnevesi and simulated natural photoperiod. Fish were fed by hand daily with commercial fish food (Biomar®; Aqualife, Aarhus, Denmark). After 4 days of acclimatization in aquaria, minnows were infected with glochidium larvae of *A. anatina* originated from Lake Koijärvi, Finland (62° 89′ N, 29° 20′ E). To perform the infection, water flow was turned off, extra aeration was provided, and 200 mL of glochidium suspension containing a total of ca. 82.000 glochidia, obtained by dissecting the marsupial gills of three female mussels, was added to aquaria. In three randomly assigned high-infection aquaria, a 45-min exposure to glochidia was applied. In three low-infection aquaria, the exposure time was 1 min. At the end of exposure, the fish were moved to new aquaria. During the experimental infection, males did not express color ornamentation, because cold water effectively prevents male color ornament expression (see Kekäläinen et al. 2011). In other words, even if at least part of the investments on colouration may have been made prior to infection, the developing colouration (see below) could still be expected to yield up-to-date information about the current condition of its carrier. Three days after experimental infections, a random sample of minnows (n = 6 and 3, in high-infection and low-infection group, respectively) from each of the six aquaria was sacrificed and their glochidia numbers were determined.

### Breeding ornamentation measurements

After the infection, water temperature was gradually raised to 18°C and layer of 20–40 mm diameter gravel was added to the bottom of the containers on 9 June 2011 (14 days after infection). This immediately activated breeding behavior of the minnows, upon which the sperm quality measurements were started. Only males that showed clear signs of breeding colouration were selected for sperm analyses. Completely nonornamented males were excluded as such individuals normally are either immature or not ready to spawn. Thus, the total number of measured males (see below) was 26 and 22 in high-infection and low-infection groups, respectively. To control for potential time effects on breeding ornamentation and sperm quality, individual males were always picked up sequentially from all six aquaria. Then, each male minnow was killed with an overdose (200 mg·L^−1^) of tricaine methanesulfonate (MS-222, Sigma®; Sigma, St. Louis, MO). Immediately after this, digital photographs of the male abdominal coloration were taken in the standard illumination by using Olympus digital camera (SP-500UZ, Olympus Corp., Tokyo, Japan). Later Hue, Saturation, and Lightness (HSL color coordinates) of the abdominal colouration were analyzed by using Adobe Photoshop CS4. Colouration was measured in two standardized areas: A rectangle with the four corners defined by the origins of the pectoral fins and the end of the gill covers, and a rectangular area between the origin and tip of the left and right ventral fins (Kekäläinen et al. 2011). Average HSL values of these two areas were used in the statistical analyses.

### Sperm quality measurements

After photographing males’ milt was stripped on petri dishes and their sperm quality was determined by using Computer Assisted Sperm Analysis, CASA (Integrated Semen Analysis System, ISAS v1: Proiser, Valencia, Spain) with B/W CCD camera (capture rate 60 frames s^−1^) and negative phase contrast microscope (200× magnification). When fish stripping did not yield enough sperm for CASA, we dissected milt directly from the testes (n = 36 and 12 for stripped and dissected males, respectively). Potentially biasing effect of the sperm collection method was controlled by including it as a fixed factor on the statistical models (see below). Prior to measurements, all milt samples were diluted to 0.9% NaCl solution. Sperm motility analyses were performed by adding 0.1 μL of sperm dilution to Leja® 2-chamber (chamber height 20 μm, volume 6 μL) microscope slides (Leja, Nieuw-Vennep, The Netherlands) and by activating sperm with 3 μL of Lake Konnevesi water. Sperm motility parameters were measured 10 s after activation (three replicate measurements/male). All sperm measurements were performed blind, that is, without knowing the infection status of the males. Measured sperm quality parameters were (1) straight line velocity (VSL); (2) curvilinear velocity (VCL); (3) average path velocity (VAP); (4) straightness of the swimming trajectory (STR); (5) linearity of the swimming trajectory (LIN); and (6) the percentage of static (immobile); and (7) rapid sperm cells. All experiments were performed according to the license of the Finnish Animal Experiment Board (ESLH-2008-10038/Ym-23).

### Fish and gonad size, breeding tubercles and parasitological examination of the males

After the colouration and sperm measurements, fish fresh mass (to 0.1 g) and total length (to 0.1 cm) were determined and their breeding tubercles on the head were counted. Then, male gonads were dissected under the microscope and their fresh mass was measured (to 0.0001 g). Linear regression residuals from the regression between log-transformed body and gonad masses were used as a measure of gonad size differences (Tomkins and Simmons 2002). Finally, the number of *A. anatina* glochidium larvae (in the fins, gills, and the total abundance) and observed other parasites, *Neoechinorhynchus rutili* (Acanthocephala), and *Gyrodactylus* spp. (Monogenea) were determined. The abundance of the *N. rutili* and *Gyrodactylus* sp. did not differ between low-infection and high-infection group (*P* > 0.05, for both parasites).

### Statistical analyses

To reduce the number of correlated sperm variables, we conducted principal component analyses (PCA) for the measured seven parameters. PCA yielded two principal components (with eigenvalue > 1) (Table 1). The first component (PC1) described sperm motility: Swimming velocity (VSL, VCL and VAP; measurements 1–3) and proportion % of static and rapid cells (measurements 6–7), whereas the second component (PC2) was associated with the curvature (form) of the swimming trajectory (STR and LIN; measurements 4–5). The effect of experimental glochidium infection on these two sperm components and the association between male ornamentation and sperm components was tested using ANCOVAs. In the models above-mentioned, two Principal components were used as dependent factors, fish group (low infection vs. high infection) and sperm collection method (stripped vs. dissected) as fixed factors and the saturation of the male abdominal colouration and breeding tubercle number as covariates. The colouration, breeding tubercle number, male size, and gonad size differences (dependent variables) between low-infection and high-infection groups (fixed factors) were tested using one-way ANOVAs. All the statistical analyses were performed with SPSS, version 19.0 (IBM Corp., New York, NY).

**Table 1:** Results of Principal component analysis (PCA) for the European minnow sperm quality (PC1, “motility”) and curvature of the sperm swimming trajectory (PC2, “trajectory”). Highest loadings are indicated in boldface

Measurement	PC1 (motility)	PC2 (trajectory)
VSL	**0.889**	0.420
VCL	**0.982**	−0.006
VAP	**0.976**	0.127
% static	**−0.914**	−0.006
% rapid	**0.975**	0.023
LIN	0.210	**0.965**
STR	−0.060	**0.986**
Eigenvalues	4.54	2.10
% of variance	64.9	29.9
Cumulative %	64.9	94.8

VSL, straight line velocity; VCL, curvilinear velocity; VAP, average path velocity; % static, proportion of immotile cells; % rapid, proportion of rapid cells; LIN, linearity of the swimming trajectory (VSL/VCL); STR, straightness of the swimming trajectory (VSL/VAP).

## Results

### Group-specific differences in measured parasite traits, body size, and gonadosomatic index

Prevalence of infection (proportion of infected individuals) 14 days postinfection was statistically significantly higher among the high-infection group (24 out of 26, 92.3%) as compared to low-infection group (11 out of 22, 50.0%) (*χ*^2^ = 10.801, df = 1, *P* = 0.001). Mean glochidium number in the high-infection and low-infection groups was 5.2 (±0.6 SE) and 1.2 (±0.4 SE) individuals, respectively. Thus, the mean abundance of infection was on the average over four times higher in the high-infection group (one-way ANOVA, *F*_1,47_ = 23.69, *P* < 0.001). Furthermore, the mean abundance of glochidia 3 days after infection was ca. 20 times higher in the high-infection group than in low-infection fish (31.83 ± 3.44 vs. 1.67 ± 0.33), indicating that infection-related costs have been significantly higher in highly-infected minnows. Fish of the high- and low-infection group did not differ by their fresh body mass and total length (±SE), respectively: high-infection group = 1.29 ± 0.10 g and 56.9 ± 1.39 mm; low-infection group = 1.43 ± 0.10 g and 59.05 ± 1.26 mm; one-way ANOVA, length: *F*_1,46_ = 1.49, *P* = 0.229; fresh mass: *F*_1,46_ = 1.19, *P* = 0.281). Also the average gonad size (i.e., body size-gonad size regression residuals ± SE) was similar in both groups (one-way ANOVA, *F*_1,45_ = 3.57, *P* = 0.065).

### Associations between parasite numbers, sperm quality, and ornamentation

**Figure 1:**
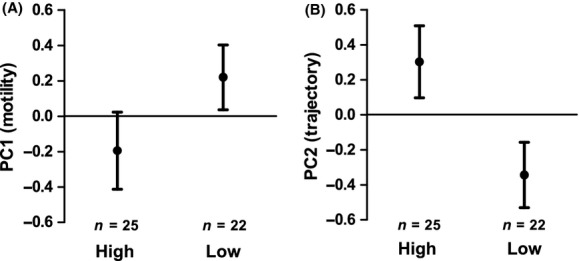
Mean (±SE) principal component score values of sperm motility, PC1 (A) and straightness/linearity of the sperm swimming tracks, PC2 (B) in high glochidia infection and low glochidia infection groups of the European minnow.

Sperm motility (swimming velocity and proportion of static and rapid cells, PC1) did not differ between low-infection and high-infection groups (*F*_1,41_ = 2.40, *P* = 0.129) (Fig. 1; Table 2), but was higher in stripped samples than when sperm was dissected from the gonads (*F*_1,41_ = 12.84, *P* = 0.001). On the other hand, if all noninfected fish were excluded from the analysis, sperm motility (PC1) was found to be lower in high-infection group (*F*_1,28_ = 7.37, *P* = 0.011). Curvature of the sperm swimming trajectory (STR and LIN, PC2) was lower in high-infection than in low-infection group (*F*_1,41_ = 4.53, *P* = 0.039), but no difference was found between sperm sampling methods (*F*_1,41_ = 1.65, *P* = 0.206). Results of PC2 remained the same also when noninfected individuals were removed from the model (data not shown).

**Table 2:** Mean (±SE) values of measured European minnow sperm parameters in the high-infection (*n* = 25) and low-infection (*n* = 22) groups

Measurement	High infection	Low infection
VCL (μm·s^−1^)	93.95 (±9.26)	111.35 (±7.12)
VSL (μm·s^−1^)	50.40 (±5.75)	52.73 (±3.60)
VAP (μm·s^−1^)	78.01 (±8.34)	88.93 (±(5.87)
LIN	52.05 (±1.95)	47.47 (±1.42)
STR	63.87 (±1.34)	59.53 (±1.41)
% Rapid sperm	42.93 (±6.48)	58.21 (±6.07)
% Static sperm	28.18 (±6.82)	9.65 (±4.05)

VSL, straight line velocity; VCL, curvilinear velocity; VAP, average path velocity; % static, proportion of unmotile cells; % rapid, proportion of rapid cells; LIN, linearity of the swimming trajectory (VSL/VCL); STR, straightness of the swimming trajectory (VSL/VAP).

**Figure 2:**
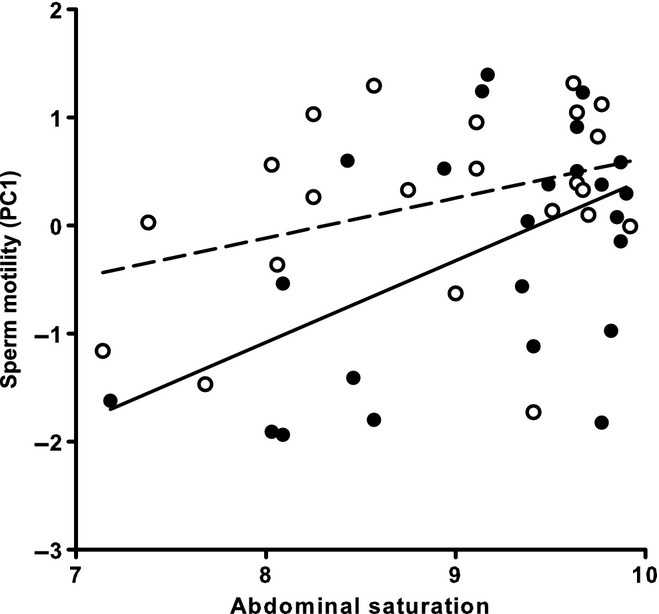
The association between male abdominal saturation (square root transformed) and sperm motility (PC1) in European minnows. Filled circles/solid line = high glochidia infection group, open circles/dashed line = low glochidia infection group.

Male abdominal saturation was positively associated with sperm motility (PC1) (*F*_1,41_ = 9.00, *P* = 0.005) (Fig. 2), but not with the curvature of the sperm swimming trajectory (PC2) (*F*_1,41_ = 1.94, *P* = 0.172). Male breeding tubercle number was not associated with sperm motility (*F*_1,41_ = 1.61, *P* = 0.211) or sperm swimming trajectory (*F*_1,41_ = 0.11, *P* = 0.742). No interaction was found between infection group and male colouration (PC1: *F*_1,38_ = 1.45, *P* = 0.237; PC2: *F*_1,38_ = 0.51, *P* = 0.480), between infection group and breeding tubercle number (PC1: *F*_1,38_ = 0.02 *P* = 0.962; PC2: *F*_1,38_ = 0.96, *P* = 0.334) nor between male colouration and breeding tubercles (PC1: *F*_1,38_ < 0.01 *P* = 0.992; PC2: *F*_1,38_ = 0.05, *P* = 0.828). Male abdominal colouration did not differ between infection groups (one-way ANOVA, *F*_1,46_ = 1.19, *P* = 0.281), but males in the high-infection group had less breeding tubercles than males in the low-infection group (one-way ANOVA, *F*_1,45_ = 4.17, *P* = 0.047).

## Discussion

We found that male minnows in the high-infection group had more straightforwardly swimming sperm and less breeding tubercles than males in the low-infection group, but that male abdominal colouration did not differ between the groups. We did not find group-specific differences in sperm motility when all the analyzed individuals were included in the model. However, sperm motility was lower in high-infection group than in low-infection group when only infected (14-days postinfection) individuals were included into model. Furthermore, in both experimental groups, saturation of the male abdominal colouration was positively associated with male sperm motility. As male gonadosomatic index and male body size did not differ between groups, sexual maturity differences and size-related sperm quality effects most likely cannot explain our results. This in turn suggests that observed differences between groups are largely dependent on differential parasite abundance.

Effective immune system and ability to reproduce successfully are closely linked traits and crucial components of animal fitness in the nature (Lawniczak et al. 2007). However, as these two processes often are strongly traded-off against each other, improved fitness in one trait should decrease fitness in the other (Tschirren and Richner 2006; Gasparini et al. 2009; Fabian and Flatt 2012). In the present study, we found that in both of our experimental infection groups the final abundance (14 days postinfection) of glochidia was relatively low. However, we also found that 3 days after infection glochidia abundance within the high infection group was ca. six times higher than the final abundance and ca. 20 times higher than in the low-infection group. As such rapid decrease in glochidia number is a direct consequency of various host immune reactions (Douda et al. 2012), infection-related costs can be expected be significantly higher in highly-infected minnows.

Present results indicate that glochidia infection may affect mainly on sperm swimming trajectory, reducing the curvature of the sperm swimming pattern. In externally fertilizing organisms, where eggs’ location is unpredictable, less straightforward, or circular movement pattern may have selective advantage, as it may prevent sperm from swimming further away from the egg before detecting the egg chemoattractant gradient (Friedrich and Julicher 2008; Liu et al. 2011). Accordingly, Fitzpatrick et al. (2012) recently showed that although sperm swimming velocity is often believed to be the most important predictor of sperm fertilization ability, in externally fertilizing marine mussel (*Mytilus galloprovincialis*) selection does not favor high swimming velocity, but sperm that have ability to swim in curved swimming trajectories. Thus, it is possible that glochidia infection reduces minnow males’ ability to fertilize the eggs, because sperm of heavily infected males may have reduced ability to locate the eggs. However, as the fertilization success of the males was not studied this hypothesis (and the mechanistic basis of this finding) remains to be clarified in the future.

We found that infection intensity did not explain variation in male abdominal colouration, but that glochidia infection may have negative impact on the other male ornament trait, the number of breeding tubercles. This partly contradicts the findings of several earlier studies that have suggested that male ornamental expression are positively associated with immunity, because only males in good condition are able to invest in ornamentation without compromising viable immune system (Rantala et al. 2000; Lawniczak et al. 2007; Kerr et al. 2010). However, Lawniczak et al. (2007) argued that expression of sexual ornamentation may be dependent on a complex balance between minimizing costs of parasitism and costs of immunity, when both positive and negative (or nonexistent) associations between immunity and ornamental expression are equally possible. Male breeding tubercle number in minnows has earlier been shown to be positively associated both with male dominance and fertilization success (Jacob et al. 2009). Thus, reduced number of breeding tubercles in high-infection group could indirectly indicate that glochidia infection decrease competitive ability and reproductive success of the males.

In addition to increasing immunity-related costs, parasites can also have number of other effects on host fitness, including increased mortality, reduced growth rate, and reduced reproductive success (Khokhlova et al. 2002; James et al. 2008; Gooderham and Schulte-Hostedde 2011). Parasites can also increase male autoimmune reactions against own sperm cells, as immune system upregulation during pathogen infections may weaken sperm protection (immunosuppression) against these reactions (Meinhard and Hedger 2011). Parasites can also directly damage the host or decrease its nutrient resources (Sheldon and Verhulst 1996; Zuk and Stoehr 2002; Schwanz 2008; Gooderham and Schulte-Hostedde 2011). Thus, it is possible that the observed effects of glochidia infection on minnow reproductive traits cannot be explained exclusively by elevated immunity costs. However, as we did not study the costs of immune responses per se, the relative importance of parasite-induced (direct) costs and immunity-related (indirect) costs and potential effect of abovementioned autoimmune reactions on minnow sperm quality and ornamental expression remains to be determined in further studies. Furthermore, although our experimental groups had clearly differential mean infection abundances and thus also trade-off costs, in the future studies the strength of the above-mentioned trade-offs should be determined also for completely parasite-free fish.

Life-history theory predicts that investments into elaborate secondary sexual ornamentation have negative impact on other reproductive traits, which is expected to result in negative association between ornamental expression and sperm quality (Parker and Pizzari 2010; Simmons et al. 2011; Parker et al. 2013). However, the direction of association may be also highly dependent on genetic variation in both resource acquisition and allocation (Van Noordwijk and de Jong 1986). Negative associations among reproductive traits are expected only when variation in male resource acquisition ability is smaller than variation in resource allocation. However, in opposite situations (resource acquisition > resource allocation) associations are expected to be positive.

Supporting one of the predictions of phenotype-linked fertility hypothesis (see Pilastro et al. 2008), we found that ornamental expression was positively associated with sperm motility in both experimental groups (high and low infection). In other words, in both groups males with more saturated abdominal colouration have higher quality sperm than their less saturated conspecifics. Besides indicating individual differences in resource acquisition and allocation abilities, this finding also suggest that infection-related costs may not create strong trade-off between ornamental expression and sperm quality even when males have invested more resources on ornamentation (highly ornamented males). This in turn suggests that: (1) Reproduction-related costs of glochidia infection may be asymmetrical and thus reduce fitness only in a subset of reproductive traits (sperm quality and breeding tubercle number), whereas costs to other traits (e.g., abdominal colouration) are minor and/or that (2) resources in our experimental conditions did not severely constrain simultaneous investments in several life-history traits. Supporting latter explanation all the experimental fish were fed daily with nutrient rich commercial fish food, which may have allowed high investments both in bright ornamentation and high quality sperm (Spitze et al. 1991; Reznick et al. 2000).

In conclusion, our results show that glochidia infection may reduce sperm ability to locate the eggs and male competitive ability (signaled by breeding tubercles), which together may have negative impact on male fertility. Male abdominal colouration did not differ between experimental groups, but in both groups, male breeding ornamentation predicted male sperm motility. This suggests that brightly colored males may not risk their sperm quality by allocating resources on elaborate ornamentation and thus also that glochidia infection may not create strong trade-off between color expression and sperm quality. This indicates that reproduction-related costs of glochidia infection may be asymmetrical and directed to only part of the reproductive traits and/or that when the available resources are abundant, simultaneous investments in several life-history traits may be possible even in the presence of resource trade-offs.
